# Comparative Effectiveness of Iontophoresis vs. Low Dye Taping in Plantar Fasciitis: A Systematic Review

**DOI:** 10.1007/s43465-025-01477-4

**Published:** 2025-07-03

**Authors:** Aurora Castro-Méndez, Lucía Roldán-Fernández, Natalia Tovaruela-Carrión, Manuel Pabón-Carrasco, Juan Álvarez-Cordero, María Vázquez-Castro

**Affiliations:** 1 Grupo de Investigación en Liderazgo Hermes CTS-601, Departamento de Podología, Facultad de Enfermería, Fisioterapia y Podología. Liderazgo DS-30 Bases biomédicas del pie que afectan al apoyo y la marcha, Instituto de Biomedicina de Sevilla, IBiS/Universidad de Sevilla, 41009, Sevilla, España; 2 Independent Researchers, Luis Montoto St: 91, 41018, Seville, Spain; 3 Grupo de Investigación Hermes CTS-601, Departamento de Podología, Facultad de Enfermería, Fisioterapia y Podología. Grupo de Investigación DS-30 Bases biomédicas del pie que afectan al apoyo y la marcha, Instituto de Biomedicina de Sevilla, IBiS/ Universidad de Sevilla, 41009, Sevilla, España; 4 CTS-1050 “Complex Care, Chronicity and Health Outcomes” Research Group, Faculty of Nursing, Physiotherapy and Podiatry, University of Seville, 41009, Seville, Spain

**Keywords:** Low-dye taping, Iontophoresis, Plantar fasciitis, Foot, Therapy

## Abstract

**Background:**

Plantar fasciitis (PF) is a frequent cause of heel pain, affecting approximately 10% of the population. Conservative treatments such as iontophoresis and low-dye taping (LDT) are widely used to alleviate symptoms, often providing short-term pain relief.

**Objective:**

This systematic review aims to compare the efficacy of iontophoresis (with 5% acetic acid, 0.4% dexamethasone, dexamethasone and lidocaine, or placebo) versus low-dye taping (LDT) in treating plantar fasciitis. Additionally, it evaluates the combined effect of iontophoresis and LDT application.

**Methods:**

A systematic search was conducted in Scopus, PubMed, Web of Science, CINAHL, and the Cochrane Library databases, following PRISMA guidelines and the Cochrane Handbook for Systematic Reviews of Interventions. Inclusion and exclusion criteria were predefined. Two independent reviewers screened and extracted data from eligible studies, assessing their quality. Included studies comprised randomized controlled trials, non-randomized clinical trials, case–control studies, systematic reviews, and meta-analyses. The review protocol was registered with PROSPERO (registration number: [blind for review]).

**Results:**

Eight studies published between 1997 and 2018 were included, providing a moderate level of evidence. Both iontophoresis (with the specified agents) and low-dye taping, alone or combined, were associated with statistically significant reductions in pain scores compared to baseline.

**Conclusion:**

Iontophoresis and low-dye taping are effective conservative interventions for plantar fasciitis, with their combined use also showing beneficial effects. These treatments can be considered viable options to reduce pain in patients with plantar fasciitis.

## Introduction

Plantar fasciitis (PF), also referred to as plantar fasciosis, is the most common cause of heel pain [1, 2], affecting approximately 10% of the population during their lifetime [3]. The etiology of PF is considered multifactorial and not yet fully understood, with risk factors including obesity, sedentary lifestyle, repetitive stress, physical activity, and male sex among others [4–9]. According to Mardani et al., PF is regarded as a degenerative condition (fasciosis) when symptoms persist beyond 8 weeks, with the chronic stage defined after this time frame [10, 11].

The diagnosis of PF is primarily clinical [12–14], but imaging techniques such as ultrasound, elastography, or sonoelastography can confirm characteristic plantar fascia thickening [15, 16]. Typical ultrasound findings include hypoechogenicity, increased tissue rigidity, and disruption of the normal fibrillar pattern [17, 18].

Conservative treatment is the first-line approach in managing PF, with surgical interventions reserved for cases refractory to conservative therapies [19]. Common conservative modalities include orthotics, dry needling, stretching, pharmacological therapy, manual therapy, shockwave therapy, night splints, physical therapy techniques (ultrasound, electrotherapy, phonophoresis), intrinsic foot muscle strengthening, and taping [1, 20, 21].

Corticosteroid injections are frequently used for their anti-inflammatory and analgesic effects in PF [22]. Crawford et al. reported pain relief up to one month post-injection, although effects typically diminish by three months [23]. However, injections can be painful and are not suitable for all patients, with potential adverse effects including infection, skin atrophy, and plantar fascia rupture [22].

Iontophoresis is a non-invasive technique that uses low-voltage electrical currents to deliver corticosteroids or acetic acid transdermally into soft tissues [24–26]. It is employed in approximately 13.04% of PF cases [27]. Studies have demonstrated that iontophoresis with 0.4% dexamethasone can restore function in musculoskeletal conditions and may serve as an alternative to corticosteroid injections [28]. Acetic acid (typically 5%) delivered by iontophoresis forms calcium acetate, which binds and facilitates the removal of calcium deposits, making this technique useful in PF and other calcific tendinitis conditions [29, 30].

Advantages of iontophoresis include preservation of the skin barrier and avoidance of needle-related tissue damage and pain. This minimizes risks such as plantar fascia rupture and infection commonly associated with corticosteroid injections [22].

Low-Dye taping (LDT) is a widely used taping technique designed to provide temporary external support to the medial foot arch (MFA), addressing biomechanical factors contributing to PF [31, 32]. Among various taping methods (e.g., X-arch, High-Dye), LDT is considered the gold standard and is frequently applied in early PF management [32]. Orthopedic devices combined with taping have demonstrated enhanced effectiveness compared to single modalities [27].

Despite advances in PF treatment, symptom management remains challenging. Corticosteroid injections offer transient pain relief but are associated with potential complications and lack standardized guidelines for use [19, 26]. Thus, exploring alternative or adjunctive conservative treatments is essential.

This review specifically compares iontophoresis and LDT, given their widespread use and potential complementary mechanisms—iontophoresis delivering anti-inflammatory agents directly into affected tissues and LDT providing mechanical support to the foot arch. Additionally, the combined application of both treatments is assessed, as this may offer synergistic benefits.

Therefore, this review aims to compare the efficacy of iontophoresis (with 5% acetic acid, 0.4% dexamethasone, dexamethasone and lidocaine, or placebo) versus LDT in the treatment of PF. Furthermore, it evaluates the combined effect of iontophoresis and LDT application. The guiding research question is: In the symptomatic treatment (pain reduction) of PF, which is more effective—iontophoresis, LDT, or their combined use?

## Methods

The search strategy was conducted between February and October 2021 on the databases: Scopus, PubMed, Web of Science, CINHAL, and the Cochrane Library.

This systematic review was conducted following the Preferred Reporting Items for Systematic Reviews (PRISMA) guidelines and the Cochrane Guide for Systematic Reviews of Interventions. The systematic review protocol was registered in the International Prospective Register of Systematic Reviews PROSPERO (number: ‘‘[blinded for review]”).

The research question followed the PICOS criteria (P—patients: adult population with PF; I—intervention: symptomatological treatment of PF with iontophoresis; C—comparison with LD-type bandage and with combined treatment with LDT-type bandage with iontophoresis; and O—Outcomes: pain reduction).

To design the search strategy, index terms and free text words related to keywords of the review objective were used and connected with Bolean operators (AND, OR and NOT).

The following two common search strategies were used for all databases: (‘‘Plantar fasciitis’’ OR ‘‘Heel pain’’) AND treatment AND (‘‘iontophoresis’’ OR 'dexamethasone' OR ‘‘acetic acid’’) NOT (‘‘injections’’ OR ‘‘surgery’’) and (‘‘Plantar fasciitis’’ OR ‘‘Heel pain’’) AND treatment AND 'low dye taping'. The description of the database is shown in Table 1.

**Table 1:** Search strategies used in each database

	Database	Search strategies	Results
Medline (Pubmed)	1st search	(‘‘Plantar fasciitis’’ OR ‘‘Heel pain’’) AND treatment AND (‘‘iontophoresis’’ OR ‘‘dexamethasone’’ OR ‘‘acetic acid’’) NOT (‘‘injections’’ OR ‘‘surgery’’)	8
	2nd search	(‘‘Plantar fasciitis’’ OR ‘‘Heel pain’’) AND treatment AND ‘‘Low-Dye Taping’’	5
CINAHL	1st search	(‘‘Plantar fasciitis’’ OR ‘‘Heel pain’’) AND treatment AND (‘‘iontophoresis’’ OR ‘‘dexamethasone’’ OR ‘‘acetic acid’’) NOT (‘‘injections’’ OR ‘‘surgery’’)	18
	2nd search	(‘‘Plantar fasciitis’’ OR ‘‘Heel pain’’) AND treatment AND ‘‘Low-Dye Taping’’	4
Web of Science	1st search 2nd search	(‘‘Plantar fasciitis’’ OR ‘‘Heel pain’’) AND treatment (‘‘Plantar fasciitis’’ OR ‘‘Heel pain’’) AND treatment AND (‘‘iontophoresis’’ OR ‘‘dexamethasone’’ OR ‘‘acetic acid’’) NOT (‘‘injections’’ OR ‘‘surgery’’)	15 17
	2nd search	(‘‘Plantar fasciitis’’ OR ‘‘Heel pain’’) AND treatment AND (‘‘iontophoresis’’ OR ‘‘dexamethasone’’ OR ‘‘acetic acid’’) NOT (‘‘injections’’ OR ‘‘surgery’’)	17
Scopus	1st search	(‘‘plantar fasciitis’’ OR ‘‘heel pain’’) AND treatment AND (‘‘iontophoresis’’ OR ‘‘dexamethasone’’ OR ‘‘acetic acid’’) NOT(‘‘injections’’OR ‘‘surgery’’)	5
	2nd search	(‘‘Plantar fasciitis’’ OR ‘‘Heel pain’’) AND treatment AND ‘‘Low-Dye Taping’’	17
Cochrane	1st search	(‘‘Plantar fasciitis’’ OR ‘‘Heel pain’’) AND treatment AND (‘‘iontophoresis’’ OR ‘‘dexamethasone’’ OR ‘‘acetic acid’’) NOT (‘‘injections’’ OR ‘‘surgery’’)	24
	2nd search	(‘‘Plantar fasciitis’’ OR ‘‘Heel pain’’) AND treatment AND ‘‘Low-Dye Taping’’	2

After removing duplicates (Check for duplicates of Mendeley toll), two independent reviewers (LRF and ACM) screened the title and abstract of all articles for eligibility. In case of disagreement, a consensus was reached with the opinion of a third reviewer (EFG).

The inclusion criteria for this review were as follows: studies published in English, Spanish, French, or German; involving adult participants diagnosed with plantar fasciitis (PF), either unilateral or bilateral. Eligible studies were published within the last 25 years (from 1996 to 2021) and included randomised controlled trials (RCTs), non-randomised clinical trials, case–control studies, cohort studies, systematic reviews, and meta-analyses. Studies were required to evaluate the effect of iontophoresis (using dexamethasone and/or acetic acid) in the treatment of PF. This included studies comparing iontophoresis with placebo, no treatment, Low-Dye taping (LDT), or the combined application of iontophoresis and LDT. Additionally, studies had to report outcomes using validated measurement tools such as the Visual Analogue Scale (VAS), the Maryland Foot Score (MFS), or the Foot Function Index (FFI).

The exclusion criteria were: studies involving animal models, and those comparing iontophoresis with surgical interventions, corticosteroid infiltrations, or other conservative treatment methods not under review in this study.

### Data Extraction

The review was carried out independently by two researchers; study authors (LRC, ACM). The references found were exported to the Mendeley bibliographic manager, and duplicate articles were deleted. Subsequently, the reviewers read the abstracts of the preselected researches, and finally the full texts of the articles that still met the inclusion criteria were analysed. Any discrepancy was resolved by a joint discussion and a third review. The following data were extracted: (1) first author; (2) study Title; (3) publication year; (4) type of design; (5) type of intervention; (6) method and duration of intervention; (7) sample size; (8) age of study participants; (9) rating scale(s) used; (10) main findings and conclusions.

### Data Synthesis and Risk of Bias

With the tool provided by Cochrane 'Review Manager' or 'RevMan', was assessed the risk of bias of the selected studies (version 5.4.1.was used).

### Quality Assessment

Two researchers (EFG, ACM) reviewed methodological quality based on the Critical Assessment Skills Programme and a PRISMA Declaration Checklist (Preferred Reporting Items for Systematic Reviews and Meta-Analyses). This process was carried out by the two different researchers to decrease the probability or risk of bias.

## Results

According to the guidance of PRISMA guidelines, the first search generated 70 articles, while the second generated 35 articles, and finally where added the 3 articles by snowball sampling, so the total was of 108 articles.

After using the ''check for duplicates'' option of the bibliographic manager, we discard those documents that we find in duplicate in several databases. In this way, we are left with 53 articles from the first search, 26 from the second search, and 3 of the articles obtained by snowball.

After discarding duplicates, we proceeded to make a more exhaustive selection of the 82 articles we had. To do this, we reviewed and read the titles and abstracts of all articles and proceeded to remove those that were not correctly classified by the databases. By this method, we limited the selection of articles to 23, of which the full texts were assessed so that they met the requirements of our PICO research question. After that only nine of the 23 articles fully met the selection criteria, so the remaining 14 were eliminated, among the main reasons being language and not original research articles.

**Fig. 1:**
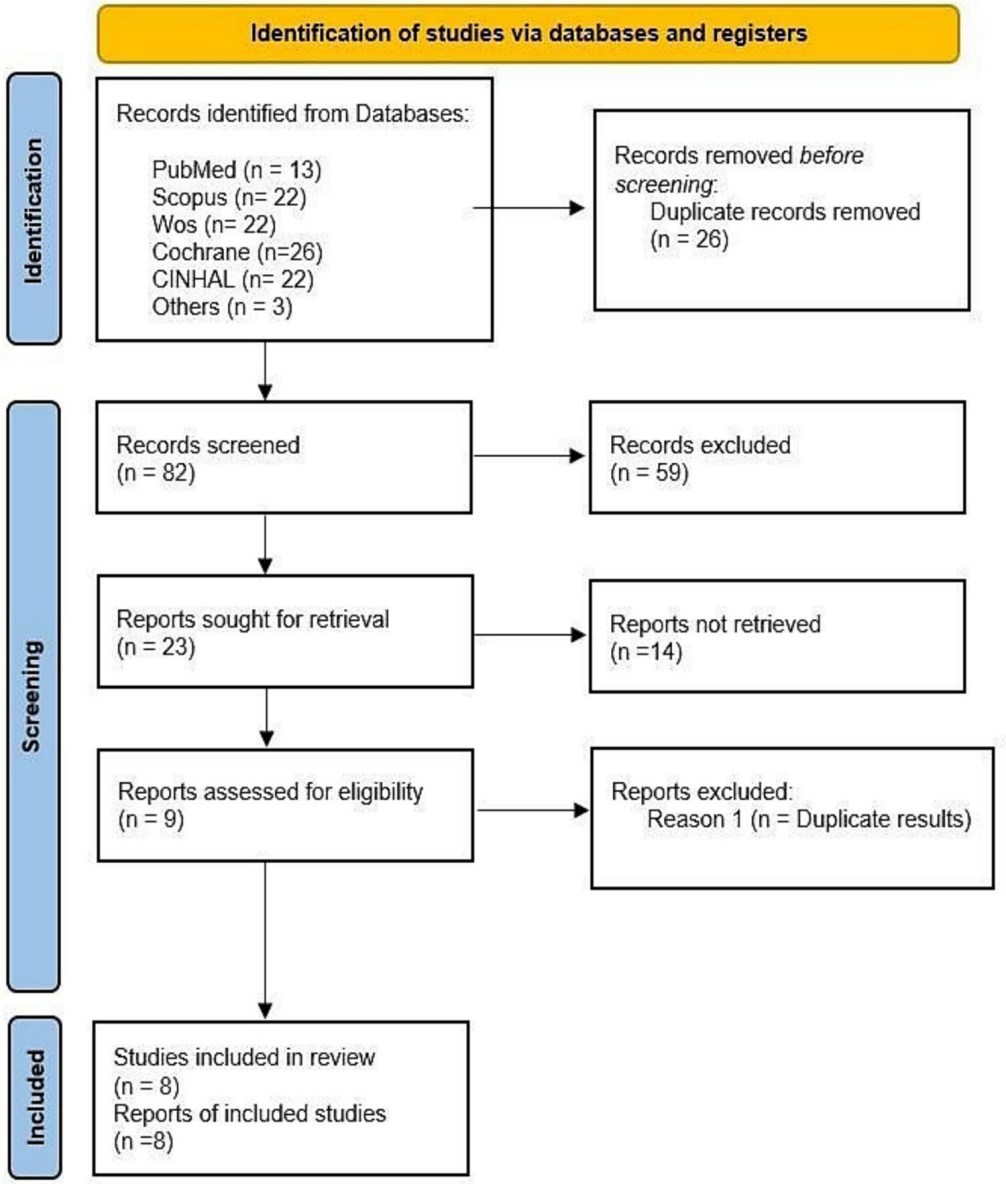
PRISMA flowchart of study selection

However, it should be noted that, after conducting an in-depth reading of the 9 articles, we could observe that 2 of them had exactly the same methodology and arrived at exactly the same results, despite being published in different scientific journals. For this reason, we included the original article and removed the duplicate study, with eight articles finalising our systematic review. Finally, the eight selected studies were analysed in detail to corroborate that their content was in accordance with the requirements previously established for the PICO research question (Fig. 1).

**Fig. 2:**
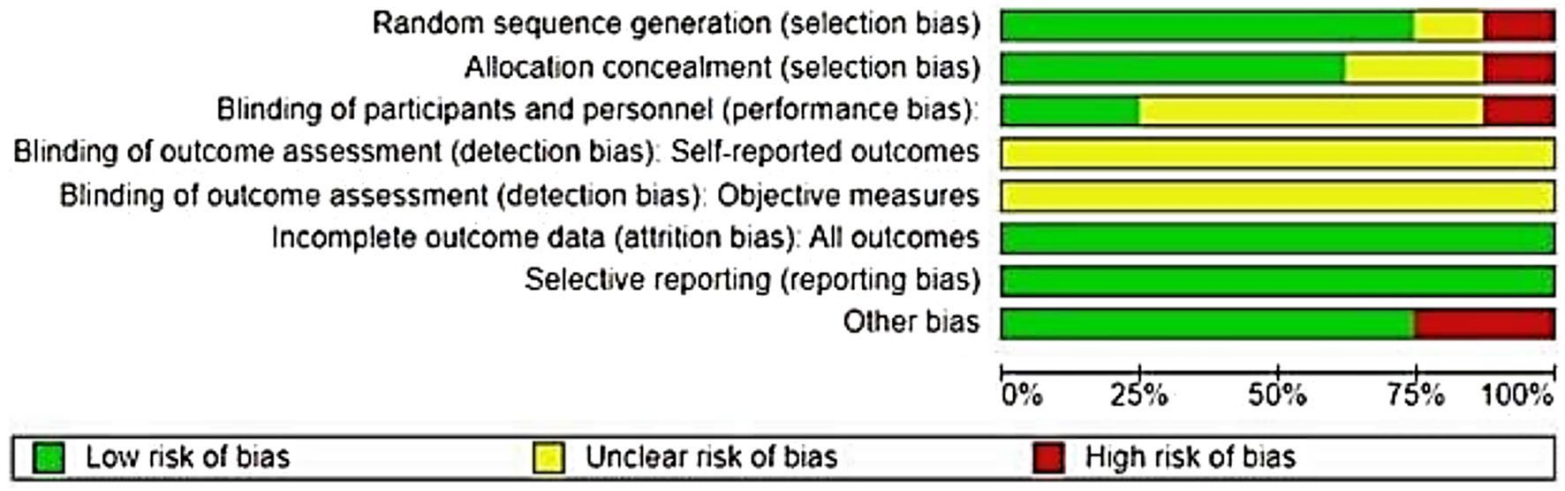
General analysis of the risks of bias in the studies analyzed

**Fig. 3:**
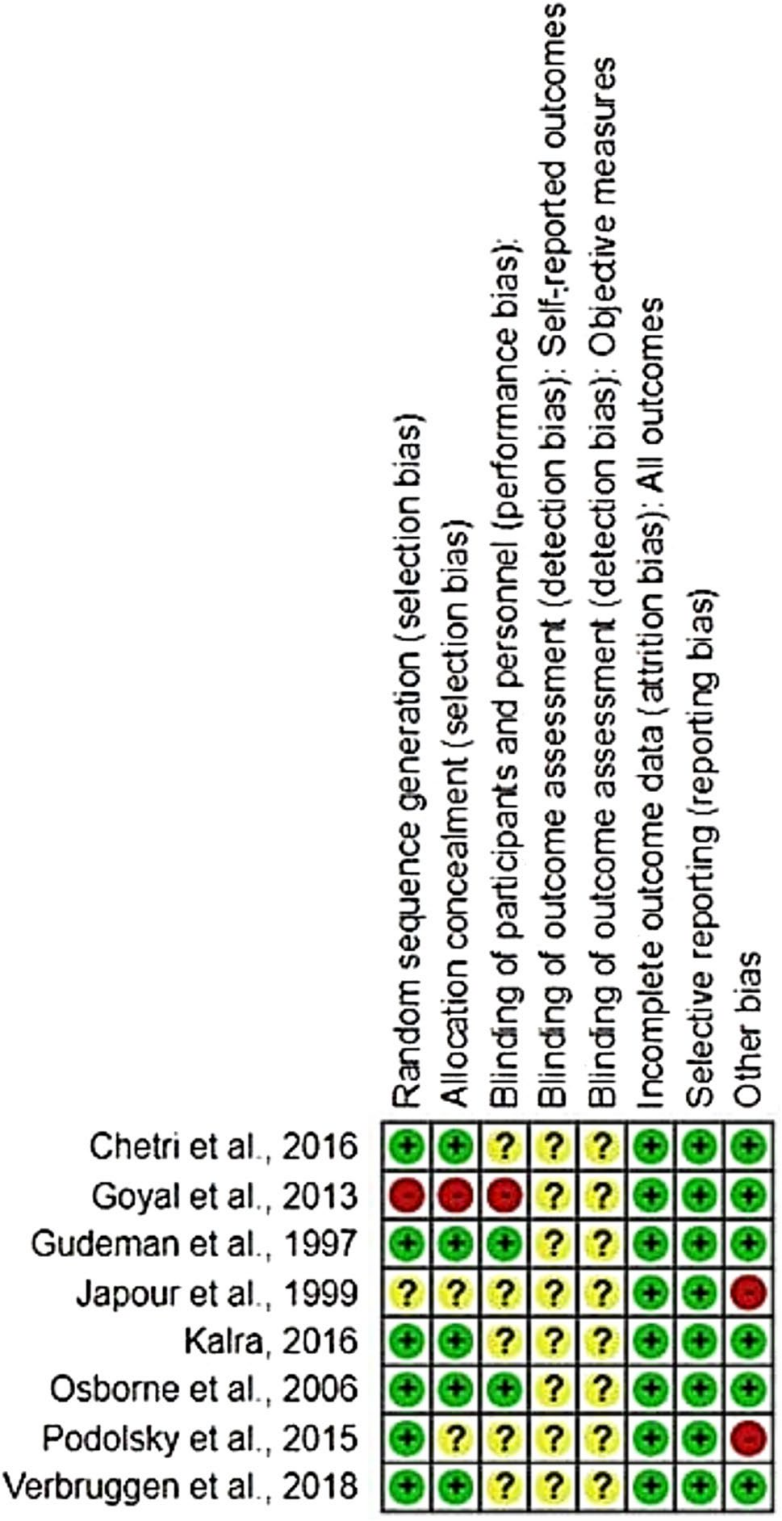
Risks of bias according to each study included in the systematic review

Risk of bias in included studies was assessed using the Review Manager software (Review Manager® or RevMan tool®), developed by The Cochrane Collaboration. This tool facilitated a structured evaluation of potential biases across studies, with results visually summarized in Fig. 2, indicating a generally low risk of bias in the majority of included publications. Furthermore, a detailed matrix diagram (Fig. 3) depicts the risk of bias levels for each study individually, categorized by color codes: green for low risk, yellow for moderate risk, and red for high risk of bias.

### Statistical Analysis of Study Quality

The CASPe and PRISMA assessment guidelines were used and applied to the eight articles included in the systematic review [33]. This process was carried out by two different researchers (LRF and ACM) to reduce the probability or risk of bias, and the score obtained is shown in Table 2.

**Table 2:** Score of each study analyzed according to the critical appraisal guides

Study	Scales	Researcher 1	Researcher 2
Gudeman et al., 1997	CASPe	9/11	9/11
Japour et al., 1999	CASPe	7/11	8/11
Osborne et al., 2006	CASPe	9/11	10/11
Goyal et al., 2013	CASPe	9/11	9/11
Chetri et al., 2016	CASPe	8/11	8/11
Kalra, 2016	CASPe	9/11	9/11
Podolsky et al., 2015	PRISMA	18/27	21/27
Verbruggen et al., 2018	PRISMA	17/27	20/27

### Results of Syntheses

Table 3 provides a comprehensive synthesis of the objectives, methodologies, results, and conclusions of all included studies. Each study is subsequently analyzed individually, with detailed descriptions of key characteristics, including methodological quality assessed using the Sackertt evidence classification system [34].

**Table 3:** Main characteristics of the studies included in this systematic review

Author/s, years	Study title	Design type	Type of intervention	Method and duration	Sample size and age	Level of evidence (Sackett	Assessment scale/s	Results	Conclusions
Gudeman et al., 1997	Treatment of Plantar Fasciitis by Iontophoresis of 0.4% Dexamethason e. A randomized, double-blind, placebo- controlled study	RCT, double blind	GC: stretching, heel support, exercises and placebo iontophoresis GExp: stretching, heel support, exercises and iontophoresis with 0.4% dexamethason e	2 weeks 6iontophoresis sessions (placebo or with dexamethason e), each lasting 20 min with a current of 40 mA	N = 39 patients (40 feet), mean age 42.1 years	Grade Ib (A)	Maryland Foot Score (measures the pain and functionality of the foot, where 100 points implies maximum independence and < 45 points great dependence	G1 had a much more significant improvement (6.8 ± 5.6 increase in the MFS scale) than the GC (3.1 ± 4.1 increase). (P = 0.022) 1-month follow-up: there was no significant difference. (P = 0.434)	The application of dexamethasone iontophoresis reduces pain and increases foot functionality in the short term (2–3 weeks) in patients with PF
Japour et al., 1999	Management of Heel Pain Syndrome with Acetic Acid Iontophoresis	Quasi- experim ental study (no control group)	Exp:iontophoresis with 5% acetic acid	3 weeks 2–3 sessions of iontophoresis with acetic acid per week of 20 min	N = 35 patients, mean age 58.6 years	Grade II-b (B)	Pain scale from 0 to 10, where 0 represents no pain and 10 represents maximum pain	Pre-treatment pain: 7.5/10 Post-treatment pain: 1.8/10	The application of iontophoresis with acetic acid reduces pain in PF
Osborne et al., 2006	Treatment of plantar fasciitis by LowDye taping and iontophoresis: short term results of a double blinded, randomized, placebo controlled clinical trial	RCT, double blind	G1: Low-Dye bandage, stretching and iontophoresis (0.4% dexamethason e) G2: Low-Dye bandage, stretching and iontophoresis (placebo) G3: Low-Dye bandage, stretching and iontophoresis (5% acetic acid)	2 weeks 6 iontophoresis sessions (with placebo, dexamethason e or acetic acid) with 40 mA current and Low-Dye bandage	N = 31 patients (42 feet), age between 18 and 75 years	Grade Ib (A)	Visual Analogue Scale (VAS), Visual Analogue Scale of stiffness and morning stiffness duration questionnaire	All groups showed significant improvements in morning pain, mean pain, and morning stiffness. However, for morning pain, the acetic acid + bandage group showed greater improvement	Low-dye taping application + iontophoresis with acetic acid, dexamethasone, or placebo reduces pain and stiffness caused by PF. For best results at 4 weeks, the bandage combined with acetic acid is preferred
Goyal et., al., 2013	Treatment of Plantar Fasciitis by Taping vs. Iontophoresis: A Randomized Clinical Trial	Quasi- experim ental study (no randomi zation of groups)	GExp: LDT and iontophoresis with 0.9% NaCl G2: LOD	1 week 1 session per day of taping + iontophoresis with NaCl with a current of 40 mA, or only bandage	N = 30 patients, age between 24 and 58 years	Grade II-b (B)	Visual analogue scale (VAS) and Foot Function Index (FFI)	After one week, there was a significant difference in VAS and FFI in both groups, but the improvement was more significant in G1 than in G2 (p < 0.05)	The combined therapy of taping and iontophoresis causes a significant decrease in pain and disability in patients with PF
Podolsky et al., 2015	Taping for plantar fasciitis	Systema tic review	The effects of various types of bandage in the treatment of PF were analyzed in 5 RCTs, 2 crossover studies and 1 repeated measures cluster study	We searched PubMed, CINAHL, PEDro, ISIWeb of Science, and Google Scholar in 2012. Studies published between 2004 and 2012 were included	N = 8 studies analyzed	Grade Ia (A)	The methodologica l quality of the studies was assessed using the PEDro rating scale	All 8 studies favored the use of different bandaging techniques. The most common technique was the LDT	The LDT has the best evidence for the treatment of PF in the short term. The long- term effect and effectiveness of this technique needs to be investigated
Chetri et al., 2016	A comparative study on effectiveness of tapping with iontophoresis and taping alone in chronic plantar fasciitis	RCT	GExp: bandage, stretching and iontophoresis with 5% acetic acid GC: bandage and stretching	2 weeks 6 treatment sessions with iontophoresis + bandage, or only bandage	N = 50 patients, age between 30 and 60 years	Grade Ib (A)	Visual analogue scale (VAS) and Foot Function Index (FFI)	The VAS and FFI scores showed a significant improvement (p < 0.05) in both groups, but greater in G1	Iontophoresis together with taping and stretching gave additional benefit compared to taping and stretching alone in reducing pain in PF
Kalra, 2016	Treatment of plantar fasciitis with dexamethason e with lidocaine hydrochloride	RCT	G1: ultrasound, stretching and iontophoresis with 0.4% dexamethason e + 4% lidocaine hydrochloride GC: ultrasound, stretching and iontophoresis with placebo	2 weeks 3 iontophoresis sessions (placebo or with dexamethason e + lidocaine hydrochloride) per week with a current of 40 mA	N = 40 patients, age: between 30 and 40 years	Grade Ib (A)	Visual analogue scale (VAS) and Foot Function Index (FFI)	G1 had a greater improvement (from 7 points to 3′17 on the EVA scale and from 44′17 to 30′17 on the FFI) than the GC (from 5.8 points to 4′40 on the EVA scale and from 43 ′40 to 37 in the FFI)	The application of iontophoresis is effective in the treatment of PF, but if it is administered with dexamethasone and lidocaine hydrochloride, it is significantly more effective
Verbruggen et al., 2018	The Effectiveness of LDT in Reducing Pain Associated With Plantar Fasciitis	Revive systemat ic vision	The effects of various types of bandaging (alone or combined with other treatments) in the treatment of PF were analyzed in 5 studies: 4 level 2 RCTs and 1 level 2 controlled clinical trial	Searches were made inPubMed, EBSCOhost, Sport Discus, Medline, and Google Scholar Articles published between 2005 and 2016 were selected	N = 5 studies analyzed	Grade Ia (A)	The methodologica l quality of the studies was assessed using the PEDro rating scale	Compared with other taping techniques for PF, LDT taping (alone or combined with other treatments) was found to be the most effective in reducing pain and increasing foot function in PF	Studies where the duration of treatment was longer produced the largest effect sizes: long-term LDT treatment may be more beneficial in reducing PF pain Current evidence supports its use as part of a multimodal intervention scheme

### Main Results

Gudeman et al. published in 1997 was the first known study in which the effects of iontophoresis with 0.4% dexamethasone were studied in the symptomatic treatment of PF were studied. The results obtained were very significant; they concluded that the application of iontophoresis with 0.4% dexamethasone reduces pain and increases foot functionality in the short term (2–3 weeks) in patients with PF, and recommended it in situations where immediate reduction in symptoms of PF was needed, for example, in elite athletes. As a limitation, this study only shows statistically significant differences in the short term (2–3 weeks), with no differences in the one-month follow-up [35]. Subsequently, Japour et al., 1999 studied the effectiveness of 5% acetic acid in reducing chronic heel pain, concluding that application of iontophoresis with 5% acetic acid reduces heel pain in the first step of the day (a characteristic sign of PF) and is therefore an effective conservative treatment for acute or recalcitrant heel pain, although it is necessary to continue investigating in this line to confirm these results. In this study, it should be noted as a limitation that it lacks a control group [36]. In 2006 Osborne et al. conducted a study randomly dividing the sample into 3 groups: Group 1 (G1) received LDT and iontophoresis with 0.4% dexamethasone as treatment, Group 2 (G2) applied LDT and iontophoresis with placebo (0.9% NaCl) and Group 3 (G3) received LDT as treatment together with iontophoresis with 5% acetic acid. Furthermore, the three groups received guidelines to stretch the leg muscle [25]. Pain and morning stiffness were measured using the Visual Analogue Scale (VAS), the Visual Analogue Stiffness Scale, and the Morning Stiffness Duration Questionnaire. These measurements were taken at the beginning of treatment, at the end of it, and after 4 weeks from the end of treatment. As a result, all groups showed significant improvements in morning pain and stiffness. However, for morning pain, the acetic acid and bandage group showed a significantly greater improvement than the rest of the groups. The researchers concluded that applying LDT together with iontophoresis and acetic acid, dexamethasone, or placebo reduces the pain and stiffness caused by PF. As indicated in the title of the study, the results shown are short-term, so the conclusions are limited to the period observed, lacking long-term results [25].

Following this line of research, Goyal et al. compared the effectiveness of LDT with the combination of this bandage and iontophoresis to reduce foot pain and increase its functionality in PF, the authors concluded that both LDT and combined bandage therapy and iontophoresis cause a significant decrease in pain and disability in patients with PF The effects of combination therapy are more beneficial. As a limitation, since it is a study with a quasi-experimental design, the study lacks randomization of groups, which increases the risk of bias and limits the strength of causal inferences. Differences between groups at baseline could influence the results [37]. In relation to combined treatments, Chetri et al. concluded that the combination of iontophoresis with acetic acid and conservative treatments can promote the recovery of foot functionality and the reduction of pain more effectively compared to the use of treatment approaches in which only one method is used. LDT is very beneficial and effective in the symptomatic treatment of PF, and acetic acid iontophoresis has shown promising results [38]. However, Kalra, 2016 concluded in his study that the application of iontophoresis with 0.4% dexamethasone and lidocaine hydrochloride shows better results in the symptomatic treatment of PF compared to iontophoresis with distilled water (placebo) [39].

Regarding the systematic review by Podolsky et al., 2015 it concluded that although several bandaging techniques have been shown to reduce pain in PF, LDT has the best evidence for the treatment of PF in the short term, although the long-term effect and effectiveness of this technique must be investigated. This review is limited by the quality of the included studies, since the individual limitations presented by the included RCTs, crossover studies, and repeated measures cluster study are transferred to the conclusions of the review [40]. Verbruggen et al. In 2018, studies in which the duration of treatment was longer (6 weeks instead of 2 or 3) produced the greatest effects, suggesting that long-term LDT treatment may be more beneficial in reducing PF pain. This review is limited by the quality of the included studies and the search parameters (2005–2016) [41].

## Discussion

Regarding plantar fasciitis (PF), pain and foot function prior to treatment indicate that, in many cases, the pain can become disabling, as shown by baseline measurements in the analyzed studies. In studies using the Visual Analog Scale (VAS) to assess pain before treatment, very similar results were found. For example, Japour et al. (1999) reported a mean pain score of 7.5 out of 10 [36], consistent with other studies showing scores above 6, 7, or even 8 [37–39].

When the Foot Function Index (FFI) was used to assess functionality before treatment, the average scores also indicated impaired function. Goyal et al. reported an initial FFI score of 41.91/90 [37], Kalra noted scores of 44.17/90 and 43.40/90 in both groups [42], and Chetri et al. reported baseline scores of 56/90 and 73/90 [38].

Regarding the symptomatic treatment of PF using iontophoresis and Low-Dye taping (LDT), studies by Japour et al., Gudeman et al., and Kalra reported statistically significant improvements (p < 0.05) in pain reduction and foot function after applying 5% acetic acid, 0.4% dexamethasone, or dexamethasone combined with lidocaine, respectively [35, 36, 39]. In studies using the VAS, a pain reduction of 4 or more points was observed after iontophoresis, while Japour et al. reported an average 10-point improvement in the FFI [36].

It is important to note that while Japour et al. applied iontophoresis as the sole treatment, the other two studies combined it with other conservative treatments. These co-interventions may have contributed to the observed improvements. According to Martin et al. and DiGiovanni et al., stretching exercises targeting the plantar fascia or Achilles tendon can yield good results for PF [21, 43].

Another key limitation across studies is the lack of consistency in the substances applied via iontophoresis, both in treatment and placebo groups. Gudeman et al. used phosphate-buffered saline as a placebo [35], while Kalra used distilled water [39]. Some studies even used saline as an active agent based on its proposed sclerotic effect on plantar fascia tissue, as described by Droog et al.

Regarding active substances, Gudeman et al. applied 0.4% dexamethasone, Kalra combined it with 4% lidocaine, and Japour et al. used 5% acetic acid [35, 36, 39]. This lack of standardization limits the ability to identify the most effective drug for pain reduction.

Among these, Gudeman et al. conducted the study with the highest level of methodological rigor, demonstrating that iontophoresis is effective in reducing pain and improving function, as measured by the MFS scale [35].

In terms of bandaging, the systematic reviews (SRs) analyzed assessed the effectiveness of different taping techniques—including LDT—as short-term symptomatic treatments for PF, sometimes combined with stretching, ultrasound, transcutaneous electrical nerve stimulation (TENS), or cryotherapy. Studies using VAS and/or FFI reported that LDT reduced pain by an average of 4.2 points, 3.17 points, and only 1.27 points in Radford et al. [44–46]. These values are comparable to the average 4-point reduction observed in iontophoresis studies, suggesting a similar level of effectiveness.

Regarding functionality, Vishal et al. found no significant differences between the taping techniques compared, although both groups improved. In contrast, Japour et al. showed a 10-point FFI improvement with iontophoresis alone, confirming the effectiveness of acetic acid in this context [36].

Podolsky et al. concluded that the long-term effects of LDT require further investigation [40]. Meanwhile, Verbruggen et al. found that longer treatments (3–6 weeks instead of 1 week) yielded larger effect sizes [41].

Concerning the combined treatment of PF with iontophoresis and LDT, three key studies were identified: Goyal et al., Osborne et al., and Chetri et al. [25, 37, 38]. Goyal compared LDT alone versus LDT plus iontophoresis with 0.9% NaCl; Chetri examined LDT alone versus LDT plus iontophoresis with 5% acetic acid; and Osborne compared three groups receiving iontophoresis with acetic acid, NaCl, or dexamethasone, all combined with LDT and stretching [25, 37, 38]. Despite differences in substances, all studies used VAS and FFI (except Osborne, who used a morning stiffness questionnaire).

Osborne et al., the study with the highest level of evidence, showed significant improvements in all groups, with acetic acid being the most effective for reducing morning pain [25]. Chetri reported a 6-point reduction on the VAS with the combined treatment, compared to 4 points with LDT alone [38]. Goyal also found that LDT combined with NaCl iontophoresis was more effective than LDT alone [37].

Comparing these studies, it can be concluded that the combination of LDT with acetic acid iontophoresis is more effective for PF pain than the combination with NaCl. Both approaches showed statistically significant improvements (p < 0.05) in pain and function, with the best results from acetic acid [37, 38].

Researchers such as Droog et al. suggest that 0.9% NaCl has a sclerotic effect, which may reduce excessive scar tissue. This supports its therapeutic use via iontophoresis, as seen in the improvements reported by Goyal et al. and Osborne et al. [25, 37]. However, the best outcomes were achieved with 5% acetic acid.

Moreover, both Osborne et al. and Gudeman et al. demonstrated that 0.4% dexamethasone applied via iontophoresis significantly reduces pain and improves function in PF [25, 35]. Kalra also found positive outcomes using dexamethasone combined with lidocaine (p < 0.05) [42].

A major limitation of this systematic review is the heterogeneity among the included studies. Despite PF being a common clinical condition, literature on iontophoresis, LDT, and their comparison remains scarce. As such, all relevant studies were included, even though they varied in iontophoresis solutions and taping techniques. This heterogeneity poses a challenge to the validity and generalizability of the findings, as treatment differences may influence outcomes and hinder direct comparisons. Nonetheless, efforts were made to minimize bias by selecting studies with similar treatment parameters.

Despite these limitations, this review offers valuable insights into a prevalent and difficult-to-manage condition. Exploring conservative therapeutic options is essential, especially in cases where recovery is delayed or when temporary treatment is needed before implementing a more definitive solution, such as orthotics. The originality and clinical relevance of this study highlight the need for continued research to strengthen the scientific evidence in this area.

## Conclusion

Iontophoresis and low-dye taping are effective conservative treatments for reducing foot pain in adults with plantar fasciitis. However, evidence from this review suggests that the combined use of iontophoresis and LDT provides superior pain relief compared to either treatment alone. Among the substances used, 5% acetic acid appears to be the most effective when applied via iontophoresis.

Nevertheless, the current body of research is limited by heterogeneity in methodologies, small sample sizes, and short-term follow-up. Future studies should aim to standardize treatment protocols, use consistent outcome measures, and include larger populations with long-term follow-up to better assess the durability of therapeutic effects.

## Data Availability

The dataset supporting the conclusions of this article is available upon request to the authors.

## Abbreviations

PF: Plantar Fasciitis
LDT: Low dye tape technique
MFA: Medial foot arch
PRP: Platelet-rich plasma
AWB: Autologous whole blood

## References

[1] Donley, B. G., Moore, T., Sierra, J., Gozdanovic, J., & Smith, R. (2007). The efficacy of oral nonsteroidal anti-inflammatory medication (NSAID) in the treatment of plantar fasciitis: A randomized, prospective, placebo-controlled study. *Foot and Ankle International,**28*(1), 20–23. 10.3113/FAI.2007.0004 17257533 10.3113/FAI.2007.0004

[2] Lemont, H., Ammirati, K. M., & Ursoni, N. (2003). Plantar fasciitis: A degenerative process (fasciosis) without inflammation. *Journal of the American Podiatric Medical Association,**93*(3), 234–237. 10.7547/87507315-93-3-234 12756315 10.7547/87507315-93-3-234

[3] Hossain, M., & Makwana, N. (2011). “Not Plantar Fasciitis”: The differential diagnosis and management of heel pain syndrome. *Orthopaedics & Traumatology, Surgery & Research,**25*(3), 198–206. 10.1016/j.mporth.2011.02.003

[4] Guillen, M. L. (2013). Ortesis para el tratamiento del dolor producido por los espolones plantares del calcaneo. *Rev Esp Podol.,**3*, 122–136.

[5] Puttaswamaiah, R., & Chandran, P. (2007). Degenerative plantar fasciitis: A review of current concepts. *The Foot,**17*(1), 3–9. 10.1016/j.foot.2006.07.005

[6] McPoil, T. G., & Hunt, G. C. (1995). Evaluation and management of foot and ankle disorders: Present problems and future directions. *Journal of Orthopaedic and Sports Physical Therapy,**21*(6), 381–388. 10.2519/jospt.1995.21.6.381 7655482 10.2519/jospt.1995.21.6.381

[7] Wearing, S. C., Smeathers, J. E., & Urry, S. R. (2003). The effect of plantar fasciitis on vertical foot-ground reaction force. *Clinical Orthopaedics and Related Research,**409*(409), 175–185. 10.1097/01.blo.0000057989.41099.d8 10.1097/01.blo.0000057989.41099.d8 12671500

[8] Allen, R. H., & Gross, M. T. (2003). Toe flexors strength and passive extension range of motion of the first metatarsophalangeal joint in individuals with plantar fasciitis. *Journal of Orthopaedic and Sports Physical Therapy,**33*(8), 468–478. 10.2519/jospt.2003.33.8.468 12968860 10.2519/jospt.2003.33.8.468

[9] Fontanella, C. G., Nalesso, F., Carniel, E. L., & Natali, A. N. (2016). Biomechanical behavior of plantar fat pad in healthy and degenerative foot conditions. *Medical & Biological Engineering & Computing,**54*(4), 653–661. 10.1007/s11517-015-1356-x 26272439 10.1007/s11517-015-1356-x

[10] Dyck, D. D., Jr. (2004). Plantar fasciitis. *Clinical Journal of Sport Medicine,**14*(5), 305–309. 15377971 10.1097/00042752-200409000-00010

[11] Mardani-Kivi, M., Karimi Mobarakeh, M., Keyhani, S., et al. (2015). Treatment outcomes of corticosteroid injection and extracorporeal shock wave therapy as two primary therapeutic methods for acute plantar fasciitis: a prospective, randomized clinical trial. *Journal of Foot and Ankle Surgery,**54*(6), 1047–1052. 10.1053/j.jfas.2015.04.026 26215551

[12] García Vidal, J. A., Piñero Palazón, J. G., Baño Alcaraz, A., Sánchez Martínez, M. P., & Medina i Mirapeix, F. (2019). Valor del Test de Silfverskiöld para el diagnóstico de la fascitis plantar. *Revista Internacional de Ciencias Podológicas,**13*(1), 41–46. 10.5209/ricp.62343

[13] Powden, C. J., Hoch, J. M., & Hoch, M. C. (2015). Reliability and minimal detectable change of the weight-bearing lunge test: A systematic review. *Manual Therapy,**20*(4), 524–532. 10.1016/j.math.2015.01.004 25704110 10.1016/j.math.2015.01.004

[14] Yates, B. (2009). *Merriman’s assessment of the lower limb* (3rd ed.). Elsevier.

[15] Radwan, A., Wyland, M., Applequist, L., Bolowsky, E., & Klingensmith, H. (2016). Ultrasonography, an effective tool in diagnosing plantar fasciitis: A systematic review of diagnostic trials. *International Journal of Sports Physical Therapy,**11*(5), 663–671. 27757279 PMC5048334

[16] Baur, D., Schwabl, C., Kremser, C., et al. (2021). Shear wave elastography of the plantar fascia: Comparison between patients with plantar fasciitis and healthy control subjects. *Journal of Clinical Medicine,**10*(11), 2351. 10.3390/jcm10112351 34072045 10.3390/jcm10112351 PMC8199455

[17] Buchbinder, R., Ptasznik, R., Gordon, J., Buchanan, J., Prabaharan, V., & Faulkner, A. (2002). Ultrasound-guided extracorporeal shock wave therapy for plantar fasciitis: A randomized controlled trial. *JAMA,**288*(11), 1364–1372. 12234230 10.1001/jama.288.11.1364

[18] Wall, J. R., Harkness, M. A., & Calder, J. S. (1993). Ultrasound diagnosis of plantar fasciitis. *Foot & Ankle,**14*(8), 465–470. 8253440 10.1177/107110079301400807

[19] Karimzadeh, A., Raeissadat, S. A., Erfani Fam, S., Sedighipour, L., & Babaei-Ghazani, A. (2017). Autologous whole blood versus corticosteroid local injection in treatment of plantar fasciitis: A randomized, controlled multicenter clinical trial. *Clinical Rheumatology,**36*(3), 661–669. 10.1007/s10067-016-3484-6 27957618 10.1007/s10067-016-3484-6

[20] Huffer, D., Hing, W., Newton, R., & Clair, M. (2017). Strength training for plantar fasciitis and the intrinsic foot musculature: A systematic review. *Physical Therapy in Sport,**24*, 44–52. 10.1016/j.ptsp.2016.08.008 27692740 10.1016/j.ptsp.2016.08.008

[21] Martin, R. L., Davenport, T. E., Reischl, S. F., et al. (2014). Heel pain - Plantar fasciitis: Revision 2014. *Journal of Orthopaedic and Sports Physical Therapy,**44*(11), A1–A33. 10.2519/jospt.2014.0303 25361863 10.2519/jospt.2014.0303

[22] Torrijos, M. A. (2009). El Tratamiento De La Fascitis Plantar. *J Sport Health Res.,**1*(2), 123–131.

[23] Crawford, F., Atkins, D., Young, P., & Edwards, J. (1999). Steroid injection for heel pain: Evidence of short-term effectiveness: A randomized controlled trial. *Rheumatology (Oxford),**38*(10), 974–977. 10.1093/rheumatology/38.10.974 10534548 10.1093/rheumatology/38.10.974

[24] Young, C. C., Rutherford, D. S., & Niedfeldt, M. W. (2001). Treatment of plantar fasciitis. *American Family Physician,**63*(3), 467–474. 11272297

[25] Osborne, H. R., & Allison, G. T. (2006). Treatment of plantar fasciitis by LowDye taping and iontophoresis: Short term results of a double blinded, randomised, placebo controlled clinical trial of dexamethasone and acetic acid. *British Journal of Sports Medicine,**40*(6), 545–549. 16488901 10.1136/bjsm.2005.021758 PMC2465091

[26] Al-Boloushi, Z., López-Royo, M. P., Arian, M., Gómez-Trullén, E. M., & Herrero, P. (2019). Minimally invasive non-surgical management of plantar fasciitis: A systematic review. *Journal of Bodywork and Movement Therapies,**23*(1), 122–137. 30691739 10.1016/j.jbmt.2018.05.002

[27] López, A. M., & Carrasco, P. G. (2014). Effectiveness of different physical therapy in conservative treatment of plantar fasciitis: Systematic review. *Revista Española de Salud Pública,**88*(1), 157–178. 10.4321/s1135-57272014000100010 24728397 10.4321/S1135-57272014000100010

[28] Runeson, L., & Haker, E. (2002). Iontophoresis with cortisone in the treatment of lateral epicondylalgia (tennis elbow)—A double-blind study. *Scandinavian Journal of Medicine and Science in Sports,**12*(3), 136–142. 10.1034/j.1600-0838.2002.02142.x 12135445 10.1034/j.1600-0838.2002.02142.x

[29] Cleland, J. A., Abbott, J. H., Kidd, M. O., et al. (2009). Manual physical therapy and exercise versus electrophysical agents and exercise in the management of plantar heel pain: A multicenter randomized clinical trial. *Journal of Orthopaedic and Sports Physical Therapy,**39*(8), 573–585. 10.2519/jospt.2009.3036 19687575 10.2519/jospt.2009.3036

[30] Sotiropoulos, D. (2014). The influence of dexamethasone with lidocaine hydrochloride iontophoresis in recreational tennis players suffering from lateral elbow tendinopathy. *Journal of Novel Physiotherapy and Rehabilitation,**4*(4), 080–085. 10.17352/2455-5487.000014

[31] Newell, T., Simon, J., & Docherty, C. L. (2015). Arch-taping techniques for altering navicular height and plantar pressures during activity. *Journal of Athletic Training,**50*(8), 825–832. 10.4085/1062-6050-50.5.05 26098272 10.4085/1062-6050-50.5.05 PMC4629939

[32] Beam, J. W. (2006). *Orthopedic taping, wrapping, bracing, & padding* (4th ed., pp. 44–46). FA Davis.

[33] Urrutia, G., & Bonfill, X. (2010). Declaración PRISMA: Una propuesta para mejorar la publicación de revisiones sistemáticas y metaanálisis. *Medicina Clínica (Barcelona),**135*(11), 507–511. 10.1016/j.medcli.2010.01.015 20206945

[34] Mella Sousa, M., Zamora Navas, P., Mella Laborde, M., & Ballester Alfaro, J. J. (2012). Niveles de evidencia clínica y grados de recomendación. *Revista de la Sociedad Andaluza de Traumatología y Ortopedia,**29*(1), 59–72.

[35] Gudeman, S. D., Eisele, S. A., Heidt, R. S., Jr., Colosimo, A. J., & Stroupe, A. L. (1997). Treatment of plantar fasciitis by iontophoresis of 0.4% dexamethasone: A randomized, double-blind, placebo-controlled study. *The American Journal of Sports Medicine,**25*(3), 312–316. 9167809 10.1177/036354659702500307

[36] Japour, C. J., Vohra, R., Vohra, P. K., Garrido, E., & Laborde, J. (1999). Management of heel pain syndrome with acetic acid iontophoresis. *Journal of the American Podiatric Medical Association,**89*(10), 543–544. 10349289 10.7547/87507315-89-5-251

[37] Goyal, M., Kumar, A., Mahajan, N., & Moitra, M. (2013). Treatment of plantar fasciitis by taping vs. iontophoresis: A randomized clinical trial. *Journal of exercise science and physiotherapy,**9*(1), 34–39. 10.8376/2013/v9i1/67578

[38] Chetri, B., Ifthikar Ali, U. T., Koch, M., & Dutta, A. (2016). A comparative study on effectiveness of taping with iontophoresis and taping alone in chronic plantar fascitis. *International Journal of Physiotherapy,**3*(2), 238–241. 10.15621/ijphy/2016/v3i2/94902

[39] Kalra, S. (2016). Treatment of plantar fasciitis with dexamethasone with lidocaine hydrochloride. *International Journal of Medical Science and Public Health Research,**5*(11), 2252–2256. 10.5455/ijmsph.2016.03042016450

[40] Podolsky, R., & Kalichman, L. (2015). Taping for plantar fasciitis. *Journal of Back and Musculoskeletal Rehabilitation,**28*(1), 1–6. 24867905 10.3233/BMR-140485

[41] Verbruggen, L. A., Thompson, M., & Durall, C. J. (2018). The effectiveness of low-dye taping in reducing pain associated with plantar fasciitis. *Journal of Sport Rehabilitation,**27*(1), 94–98. 27705070 10.1123/jsr.2016-0030

[42] Strassburger Lona, K., Hernández Porras, S., & Barquín, S. E. (2014). *Lesión Medular: Guía para manejo integral del paciente con LM crónica* (pp. 1–161). Aspaym.

[43] DiGiovanni, B. F., Nawoczenski, D. A., Lintal, M. E., et al. (2003). Tissue-specific plantar fascia-stretching exercise enhances outcomes in patients with chronic heel pain: A prospective, randomized study. *Journal of Bone and Joint Surgery,**85*(7), 1270–1277. 10.2106/00004623-200307000-00013 12851352

[44] Radford, J. A., Burns, J., Buchbinder, R., Landorf, K. B., & Cook, C. (2006). The effect of low-dye taping on kinematic, kinetic, and electromyographic variables: A systematic review. *Journal of Orthopaedic and Sports Physical Therapy,**36*(4), 232–241. 10.2519/jospt.2006.36.4.232 16676873 10.2519/jospt.2006.36.4.232

[45] Bagewadi, V., Metgud, S., & Ganesh, B. (2014). Effectiveness of plantar fasciitis taping and calcaneal taping in plantar heel pain: A randomized clinical trial. *The Indian Journal of Physiotherapy & Occupational Therapy,**4*(3), 86–90.

[46] Landorf, K. B., Radford, J. A., Keenan, A. M., & Redmond, A. C. (2005). Effectiveness of low-dye taping for the short-term management of plantar fasciitis. *Journal of the American Podiatric Medical Association,**95*(6), 525–530. 10.7547/0950525 16291843 10.7547/0950525

